# Evaluating the Use of Vibrational Spectroscopy to Detect the Level of Adulteration of Cricket Powder in Plant Flours: The Effect of the Matrix

**DOI:** 10.3390/s24030924

**Published:** 2024-01-31

**Authors:** Shanmugam Alagappan, Siyu Ma, Joseph Robert Nastasi, Louwrens C. Hoffman, Daniel Cozzolino

**Affiliations:** 1 School of Agriculture and Food Sustainability, The University of Queensland, St. Lucia Campus, Brisbane, QLD 4072, Australia; s.alagappan@uq.net.au (S.A.); siyu.ma@uq.net.au (S.M.); j.nastasi@uq.edu.au (J.R.N.); 2 Queensland Alliance for Agriculture and Food Innovation (QAAFI), Centre for Nutrition and Food Sciences, The University of Queensland, St. Lucia Campus, Brisbane, QLD 4072, Australia; louwrens.hoffman@uq.edu.au

**Keywords:** insect, flour, infrared, NIR, MIR, classification

## Abstract

Edible insects have been recognised as an alternative food or feed ingredient due to their protein value for both humans and domestic animals. The objective of this study was to evaluate the ability of both near- (NIR) and mid-infrared (MIR) spectroscopy to identify and quantify the level of adulteration of cricket powder added into two plant proteins: chickpea and flaxseed meal flour. Cricket flour (CKF) was added to either commercial chickpea (CPF) or flaxseed meal flour (FxMF) at different ratios of 95:5% *w*/*w*, 90:10% *w*/*w*, 85:15% *w*/*w*, 80:20% *w*/*w*, 75:25% *w*/*w*, 70:30% *w*/*w*, 65:35% *w*/*w*, 60:40% *w*/*w*, or 50:50% *w*/*w*. The mixture samples were analysed using an attenuated total reflectance (ATR) MIR instrument and a Fourier transform (FT) NIR instrument. The partial least squares (PLS) cross-validation statistics based on the MIR spectra showed that the coefficient of determination (R^2^_CV_) and the standard error in cross-validation (SECV) were 0.94 and 6.68%, 0.91 and 8.04%, and 0.92 and 4.33% for the ALL, CPF vs. CKF, and FxMF vs. CKF mixtures, respectively. The results based on NIR showed that the cross-validation statistics R^2^_CV_ and SECV were 0.95 and 3.16%, 0.98 and 1.74%, and 0.94 and 3.27% using all the samples analyzed together (ALL), the CPF vs. CKF mixture, and the FxMF vs. CKF mixture, respectively. The results of this study showed the effect of the matrix (type of flour) on the PLS-DA data in both the classification results and the PLS loadings used by the models. The different combination of flours (mixtures) showed differences in the absorbance values at specific wavenumbers in the NIR range that can be used to classify the presence of CKF. Research in this field is valuable in advancing the application of vibrational spectroscopy as routine tools in food analysis and quality control.

## 1. Introduction

The nutritional value of plant proteins depends on their botanical origin, as well as technological processes or transformations, which can be determined by either the use of biological (e.g., enzymatic treatment) or physical methods (e.g., extrusion) [1,2,3]. In addition to the nutritive value and amino acid content, the digestibility of the protein is also an important quality parameter that is considered when evaluating the quality of different sources of alternative proteins [4,5].

Edible insects have been recognised as an alternative food or feed ingredient due to their high protein content for both humans and domestic animals [1,2,3,4,5]. The use of these ingredients in both foods and feeds has increased due to their unique chemical composition, nutritive value, and functional properties [5,6]. Edible insects can be consumed as whole insects; however, they might be rejected by the consumers, particularly as insects are usually associated with vectors of diseases and perceived as a health risk by humans [7,8]. Therefore, processing the insects (e.g., drying, milling, and extrusion) into flour, powders, or pellets has been associated with an increase in consumer acceptability [9,10].

The utilization of insects as alternative sources of protein has increased, where the number of research and development projects on these food ingredients has been steadily growing in recent years [9,10,11]. It has been reported that edible insects have been used as ingredients in wheat bread fortification (e.g., mealworms, buffalo worms, and crickets) [9,10,11] and as functional ingredients to improve the biological and nutritional value of traditional and gluten-free breads, as well as to increase the protein content of muffins [9,10,11,12]. It has been also reported that the incorporation or mixing of insect flour with cereals and other starchy ingredients can create characteristic aroma notes or improve the texture of the foods, such as increasing the hardness of the end product or improving the consistency of the flour [9,10,11,12]. However, insect flour can also contain chitin and chitosan that can contribute positively to inhibiting the growth of some microorganisms present in the food matrix, although the presence thereof could result in the over analysis of the protein content [9,10,11,12]. In recent years, the consumers’ concerns about the safety and origin of the food ingredients and food products have intensified due to several issues that have disrupted the food supply and value chains; one such issue was the adulteration of milk powder with melamine in China to increase the protein content of milk powder [13,14]. Consequently, the need to develop and implement systems to guarantee the safety of foods is necessary for both the consumers and the food manufacturing industry. Several techniques and methods have been evaluated or proposed by several research groups to address these issues.

It is well known that the estimation of the proximate composition (e.g., proteins, starches, and carbohydrates) of a food ingredient or product provides limited information about the quality as well as other characteristics of the food, including its safety [13,14,15,16]. Additionally, the determination of the proximate composition of a food ingredient or product does not necessarily reflect the actual level of, for example, protein, which may be enhanced with insect protein (containing high levels of chitin), resulting in the adulteration or contamination of the sample [13,14,15,16]. The other techniques used by the food manufacturing industry to assess the composition of this type of foods including high-performance liquid chromatography (HPLC), gas chromatography (GC), mass spectrometry (MS), or even the use of sensory analysis [13,14,15,16].

Vibrational spectroscopy methods, such as near- (NIR) and mid- (MIR) infrared and Raman spectroscopy, have been recognized by the feed and food industries as practical analytical methods, as they provide the users and consumers with not only with a fast and inexpensive method, but also with a non-destructive alternative to monitoring and quantifying composition, functionality, and safety (e.g., adulteration, contamination, and fraud) [16,17,18,19,20]. As mentioned, food safety and security have become an important topic for consumers, as the addition of contaminants to foods, exacerbated by food fraud issues, can have important health and nutrition implications for humans [16,17,18,19,20]. Both the feed and food manufacturing industries are also interested in guaranteeing the safety of the feeds and food ingredients, as well as their products [16,17,18,19,20]. This is of especial importance in the current market, as the demand for alternative sources of proteins has increased recently.

A significant advantage of vibrational spectroscopy techniques is the ability to target food safety concerns along the supply and value chains [16,17,18,19,20]. These techniques have been used to detect and quantify the presence of contaminants, such as allergens, mycotoxins, pesticides, and heavy metals, and as traceability tools to monitor the fraudulent provenance of different plant protein ingredients and products [16,17,18,19,20]. Overall, ensuring the safety of both the alternative source- and plant-based protein foods is crucial to prevent adverse health effects and maintain consumer confidence in this type of staple food [16,17,18,19,20].

The utilization of vibrational spectroscopy (e.g., MIR and NIR) to evaluate and detect the presence of insects’ contaminants either as whole insects or fragments in cereals, such as wheat, rice, and sorghum, is not new [21,22,23,24]. More recently, infrared (IR) spectroscopy has been reported to assess and quantify the content of protein in insect-based energy bars [25].

Both MIR and NIR spectroscopy provide reliable and efficient means of monitoring and evaluating the occurrence of potential adulteration or contamination throughout the supply chain, thereby contributing to maintaining the integrity of the supply chain [26,27,28,29,30]. The use of these techniques allows for the determination of spectral fingerprints or signatures that can be used for authenticating and identifying food ingredients and food products [26,27,28,29,30]. They can also be used to verify the origin of plant-based protein ingredients by preventing adulteration and ensuring that the products meet the claimed standards and labelling requirements stated by the regulators [26,27,28,29,30]. This is particularly important as the plant-based protein industry continues to expand, and there is a need to maintain transparency and trust in the market [26,27,28,29,30].

Although, both MIR and NIR spectroscopy have demonstrated their potential to assess the adulteration and contamination of different types of binary mixtures across various food ingredients and products (e.g., cereals), there is still a shortage of research that examines the influence of the matrix on the IR spectra and the classification results in mixtures of insects with different plant and cereal flours [20]. The utilization of IR spectroscopy has been widely recognized as a valuable tool by the manufacturing food industry due to its ability to provide an initial level of screening not only about the chemical composition of the samples, but also about other characteristics, including the level of intentional contamination [26,27,28,29,30]. This advantage has been explored by researchers and the food manufacturing industry along the supply and value chains, enabling more costly methods to be used more efficiently on the suspect samples. In addition, these techniques can be easily implemented by the food manufacturing industry during the processing, transport, and storage of food ingredients and products [26,27,28,29,30]. Whereas the measurement of the chemical composition of food ingredients is highly desirable to optimize the processing of alternative proteins, the utilization of IR spectroscopy as a qualitative screening tool provides the means to monitor and detect the presence of adulterants or defects, or even fraud [26,27,28,29,30]. Consequently, the incorporation of IR spectroscopy into the toolbox of analytical methods will allow for the guarantee of the composition and safety of food ingredients and products, thereby increasing efficiency and productivity in the food industry, as well as being a means to protect the consumers.

Therefore, the objective of this study was to evaluate the ability of both near- (NIR) and mid-infrared (MIR) spectroscopy to identify and quantify the level of adulteration of cricket powder added to two plant protein (chickpea and flaxseed) meal flours.

## 2. Materials and Methods

The addition of cricket flour (CKF) to either commercial chickpea flour (CPF) or flaxseed meal flour (FxMF) was evaluated. Three different batches of CKF (approx. 1 kg each) were purchased on the Australian market as cricket protein powder (NSW, Australia). In addition, three different batches of commercial CPF and FxMF (approx. 1 kg each) were also purchased from a local supermarket in the Brisbane area (Queensland, Australia). An aliquot was taken from each of the batches, where binary mixtures were made using different proportions as reported elsewhere (e.g., 100%, 90%, 80% *w*/*w*, etc.) [31]. This procedure was repeated for the three baches. The CKF contains 60% crude protein (CP), 17% crude fat (CF), and 23% total fibre (TF), while CPF contains 21% CP, 8% CF, and FxMF 18%, and 42% CP and CF, respectively. The addition of small quantities of CFK to either CPF or flaxseed flour (FxMF) was also evaluated (from 1 to 10%). In this case, mixtures of CKF with CPF and FxMF were prepared in triplicate.

FT-NIR and FT-MIR spectra of the pure and mixture samples were collected using a Bruker Tango-R spectrophotometer (average of 64 interferograms collected at a resolution of 4 cm^−1^ in the 11,550 to 3950 cm^−1^ range) and a Bruker Alpha instrument fitted with an attenuated total reflectance (ATR) platinum diamond single-reflection module (average of 24 coadded interferograms at a resolution of 4 cm^−1^ in the 4000 to 400 cm^−1^ region) (Bruker Optics GmbH, Ettlingen, Germany). The cuvettes and ATR cell were cleaned with a mixture of 70% ethanol in water (*v*/*v*) and dried with laboratory Kimwipes^®^ between sampling. Details about the spectra collection (wavenumber range and data points) can be found in the previous reports [31,32].

Both the MIR and NIR spectra were exported from the instrument to Vektor Direktor™ (Version 1.1; KAX Group, Sydney, NSW, Australia) using OPUS software (v. 8.5) for chemometric analysis. Before data analysis and classification, the NIR spectra were pre-processed using the Savitzky–Golay second derivative (second polynomial order and 21 smoothing points) [33]. Principal component analysis (PCA) and partial least squares regression analysis (PLS) were used to classify the flour mixture samples according to the level of adulteration. The PCA and PLS models were validated using cross-validation (leave one out) [34,35,36]. The PLS regression models were also evaluated by dividing the data set into calibration and validation sets using the Kennard–Stone algorithm [37]. In this study, 40 samples were selected to develop the calibration models, while the remaining 20 samples were used for validation. The K-S algorithm allows for performing data partitioning, where knowledge of the training (calibration) data set did not affect the test data set (validation), and the predictive power of the created model subsequently increased [37]. The PLS results were evaluated using the coefficient of determination in cross-validation (R^2^_cv_) and the standard error in cross-validation (SECV) [34,35,36].

## 3. Results and Discussion

### 3.1. Mid- and Near-Infrared Interpretation

**Figure 1:**
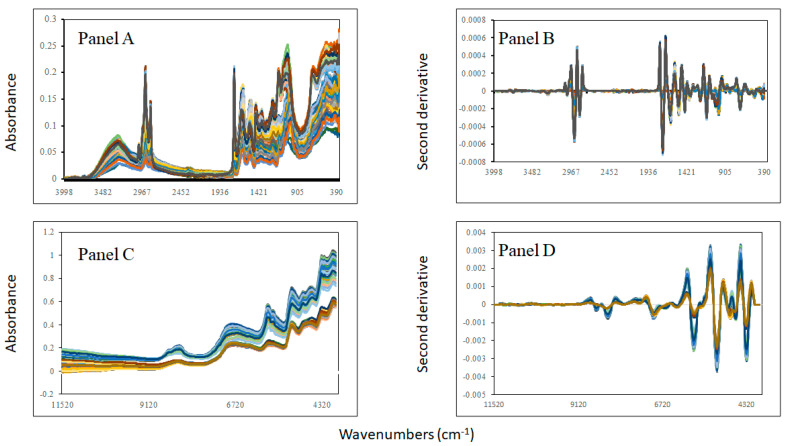
Mid-infrared raw (**Panel A**) and second-derivative spectra (**Panel B**) and near-infrared raw (**Panel C**) and second-derivative spectra (**Panel D**) of the cricket powder, chickpea, and flaxseed meal flour, and mixtures analyzed.

The spectral properties of the samples are reported in Figure 1 (MIR raw and second derivatives) (panels A and B). Differences were observed in the MIR spectra in the range between 3000 and 2700 cm^−1^ and in the fingerprint region between the 1700 and 600 cm^−1^ wavenumbers. The main variations were observed at around 3010 cm^−1^, 2919 cm^−1^, and 2851 cm^−1^, which are associated with OH stretching (around 3000 cm^−1^) and with C-H, ester groups, and the C-H_3_ of fatty acids and lipids (2919 cm^−1^ and 2851 cm^−1^) [38,39]. The region between 2900 and 2850 cm^−1^ has also been reported to be associated with the presence of chitin, a product found at various concentrations in insects [40]. In the fingerprint region, the main wavenumbers were observed at around 1742 cm^−1^, which is associated with the C=O stretching of lipids; 1624 cm^−1^ and 1517 cm^−1^ are associated with amides I and II, respectively [38,39]. The region around 1457 cm^−1^ is typically associated with lipids and proteins, whilst 1395 cm^−1^ is mainly associated with amino acid residues, 1235 cm^−1^ is associated with amide III, and that between 1100 and 900 cm^−1^ is associated with carbohydrates and polysaccharides [38,39]. In addition, in the fingerprint region between 1200 and 800 cm^−1^, these absorbances have been reported to be associated with C-O-C glycosidic linkage, COH bending, and C-C stretching vibrational modes, which are considered characteristic groups of carbohydrates, sugars, and polysaccharides, respectively [38,39]. The absorbances associated with starch can be also located between 1250 and 800 cm^−1^, which are related to the presence of the unsaturated bonds in the C=O groups primarily associated with carbohydrates, as well as unspecific CH_2_ bending vibrations, whereas those found in the latter can be attributed to vibrations arising from C-O groups, which are also observed in carbohydrates [38,39,40].

The NIR raw and second-derivative spectra of the samples analysed are shown in Figure 1, panels C and D, respectively. The main absorbances in the NIR region can be observed at around 8304 cm^−1^, which is related to fatty acids, such as oleic acid and linoleic acid; this has been reported in insects by other researchers [24,40,41,42,43,44]. The 6800 cm^−1^ wavenumber is associated with O-H groups, corresponding mainly with the water content, whilst that around 4720 cm^−1^ is associated with CONH_2_ and C=O bonded with N-H groups (e.g., peptides and proteins) [24,40,41,42,43,44]. The bands observed at around 4335 cm^−1^ and 4261 cm^−1^ are associated with the presence of aliphatic hydrocarbons (fatty acids) and lipids, respectively [24,40,41,42,43,44]. The region between 5300 and 5000 cm^−1^ is also associated with the moisture content (5600 cm^−1^) and with the N–H stretching (asymmetric) and N–H in-plane bending combination of CONH_2_ groups corresponding with proteins (5168 cm^−1^) [40,41,42,43,44]. Overall, the information in both the MIR and NIR spectra is attributed to the moisture, lipid, protein, and starch contents, as well as the concentration of chitin derived from the exoskeleton of the crickets in the samples analyzed. Therefore, due to the observed differences in both the MIR and NIR spectra of the mixtures, these data were used in combination with chemometrics to predict the level of adulteration and are reported in the following sections.

### 3.2. Principal Component Analysis

**Figure 2:**
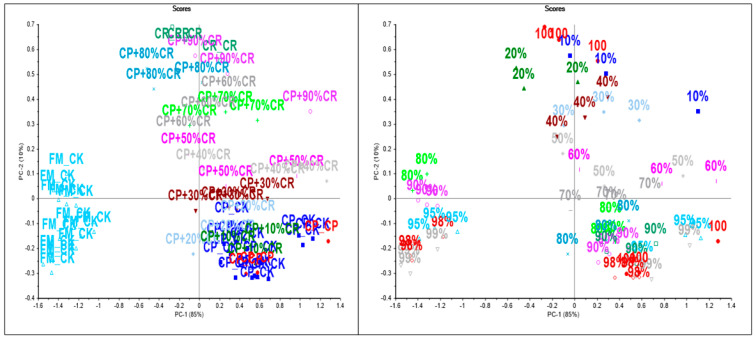
Principal component score plot of cricket, chickpea, and flaxseed meal flour mixture samples analyzed using mid-infrared spectroscopy (FM: flaxseed; CP: chickpea; CK: cricket).

**Figure 3:**
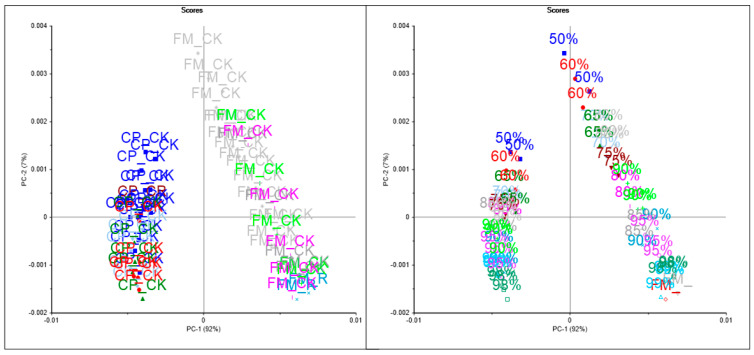
Principal component score plot of cricket, chickpea, and flaxseed meal flour mixture samples analysed using near-infrared spectroscopy (FM: flaxseed; CP: chickpea; CK: cricket).

Figure 2 and Figure 3 show the PCA score plot of the mixture samples analyzed, highlighted by mixture type (panel A) or by concentration (panel B) using either MIR or NIR spectroscopy, respectively. The two PCA score plots showed similar trends, where the main differences were associated with the type of flour (FxMF vs. CPF) along the first principal component (PC1), while the samples were clustered by concentration in the mix along PC2. Overall, both PC1 and PC2 explained 95% and 99% of the variance in the mixture samples analyzed using either MIR or NIR spectroscopy, respectively. These results highlighted the influence of the matrix on the spectral properties (MIR and NIR) of the samples analyzed. The differences between the group of flour/mixture samples can mainly be attributed by the type of flour used. In addition, the concentration or level of adulteration of the mixture or the level of cricket powder added to the plant flour (FxMF vs. CPF) influenced the cluster of samples. Separate PCA analyses were calculated for each of the binary mixtures (FxMF vs. CKF; CPF vs. CKF) using NIR spectroscopy. In these analyses, the PCA score plots showed that PC1 explained the effect of concentration in the mixtures rather the differences between the types of flour used to make the mixtures. In all the cases (both MIR and NIR spectroscopy), it can be observed that the first PC explained most of the variance in the data set associated with the addition of CKF (concentration) into the mixture.

### 3.3. Partial Least Squares Regression

To better analyse the effects of either the flour type or the concentration utilized to make the mixtures, PLS regression models were developed using all the samples (ALL), and then the mixtures of cricket and chickpea (CPF vs. CKF) and cricket and flax seed (FxMF vs. CKF) flours, separately. The PLS cross-validation statistics for the ALL samples and the binary mixtures analysed using both MIR and NIR spectroscopy after second derivative pre-processing are reported in Table 1. All the PLS models developed explained more than 90% of the variability in the data set (R^2^_CV_ > 0.90). The results based on MIR showed that the cross-validation statistics were R^2^_cv_: 0.94 and SECV: 6.68%; R^2^_cv_: 0.91 and SECV: 8.04%; and R^2^_cv_: 0.92 and SECV: 4.33% for the ALL, CPF vs. CKF, and FxMF vs. CKF mixtures, respectively. The results based on NIR showed that the cross-validation statistics were R^2^_cv_: 0.95 and SECV: 3.16%; R^2^_cv_: 0.98 and SECV: 1.74%; and R^2^_cv_: 0.94 and SECV: 3.27% for the ALL, CPF vs. CKF, and FxMF vs. CKF mixtures, respectively. Interesting, the SECV values were higher in the PLS models using MIR spectroscopy compared with those studied using NIR. Similar results were reported by other authors using ATR-MIR spectroscopy to predict the addition of crickets to whole-grain flour [20] and the contamination of insect meal in animal feeds using FT-NIR spectroscopy [24,44].

**Figure 4:**
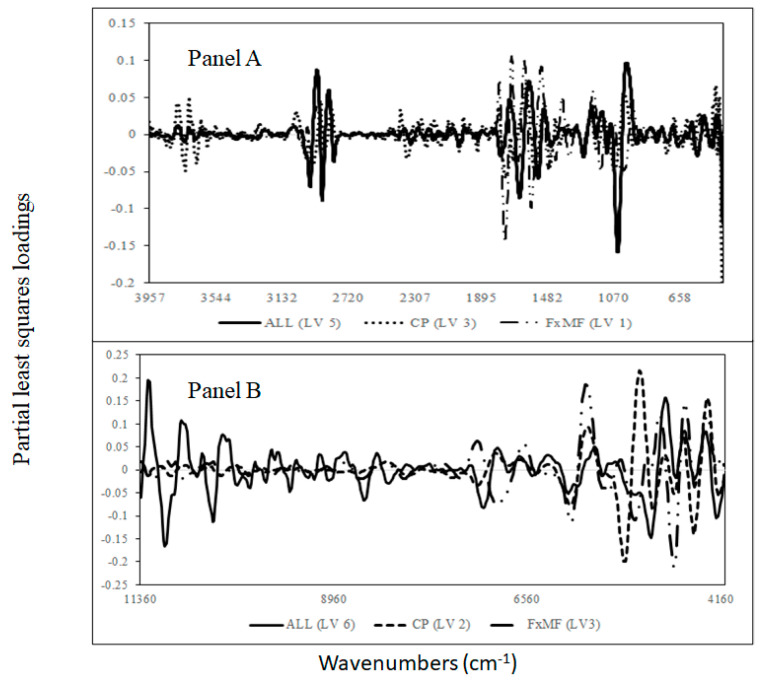
Optimal partial least squares loadings used to predict the addition of cricket powder to chickpea and flaxseed meal samples analysed using mid-infrared (**Panel A**) and near-infrared spectroscopy (**Panel B**).

Although very good cross-validation statistics were obtained, differences in the PLS loadings used by each of the models were observed. The highest and common PLS loadings in the MIR region (Figure 4, panel A) used to predict the addition of CKF into the plant samples were observed at around 2933 cm^−1^, which is associated with C-H bonds, ester groups, as well as with the C-H_3_ of fatty acids and lipids. The frequencies around 2933 cm^−1^ have also been associated with chitin. At around 1630 and 1540 cm^−1^, these frequencies are associated with the amide I and II groups of protein, respectively [38,39]. Distinctive loading was observed at around 1780 cm^−1^ in the models where CKF was added to FxMF flour.

The common and highest PLS loadings in the NIR region (Figure 4, panel B) used for the determination of the addition of cricket powder to the other flour samples were observed at around 7137 cm^−1^ and 6944 cm^−1^, with similar loading at around 5432 cm^−1^, 4440 cm^−1^, and 4256 cm^−1^. The bands at around 4335 cm^−1^ and 4261 cm^−1^ are associated with the presence of aliphatic hydrocarbons (fatty acids) and lipids [39,40,41]. While differences were observed at around 6944 cm^−1^ and 5980 cm^−1^, which are associated with the water content, the other wavenumbers, such as 5264 cm^−1^ (O-H) and 4832 cm^−1^, are associated with CONH_2_ and C=O bonded withing the N-H groups (e.g., peptides and proteins). The presence of these bands is typically associated with protein content in the sample [39,40,41,42,43,44]. The band at around 4320 cm^−1^ is associated with C-H stretching and C-H_2_ deformation, corresponding with the presence of polysaccharides, mainly the starch content [40]. The highest loadings were observed at around 5072 cm^−1^ (N-H combinations and aromatic amines) and 4512 cm^−1^ (N-H and N-H_2_ combinations), which are associated with protein and aromatic amines [40]. Although the results of this study showed that both MIR and NIR spectroscopy can detect the level of the adulteration or addition of cricket powder in CPF or FxMF flour, this study has some limitations to consider before extending its applicability. A major limitation is the number of samples analysed, as well as the limited levels of adulteration evaluated. It would be interesting to see, for example, what the lowest level of cricket powder as an adulterate could be detected by both MIR and NIR.

## 4. Conclusions

The results of this study showed the definable effect of the matrix on the PLS regression results in both the calibration statistics and the PLS loadings used by each of the models. The different combinations of flours or artificial mixtures showed differences in the absorbance values at specific wavenumbers in the MIR and NIR ranges. Nevertheless, both MIR and NIR spectroscopy could be utilized as an initial screening tool to detect the level of the addition of cricket powder to plant flour samples along the supply and value chains of alternative proteins, allowing expensive and specific methods, such as HPLC or GC-MS or DNA analyses, to be used more efficiently on suspect samples. The utilization of vibrational spectroscopy methods for the initial screening of the potential contamination of food ingredients can significantly improve the efficiency and effectiveness of quality control measures by the food manufacturing industry. Different stakeholders will benefit from the implementation of this type of approach, where the food ingredients and products can be easily and economically monitored by the food and feed manufacture industries along the supply and value chains. The implementation of these types of tools will reduce the risk of food fraud as well as guarantee both the safety and security of food ingredients and products. This type of applications will also allow for the development of effective management tools to monitor and target the adulteration or contamination of feeds and foods. Overall, research in this field is valuable in advancing the application of vibrational spectroscopy as routine tools in food analysis and quality control. However, before this type of application can be implemented, reliable models must be developed, where more samples and different types of powders and flours should be included for the proper validation of the models.

## Figures and Tables

**Table 1:** Cross-validation statistics for the prediction of the level of cricket addition to either chickpea or flaxseed flour samples analysed using mid-infrared and near-infrared spectroscopy.

	ALL	CPF	FxMF
NIR			
R^2^_CV_	0.95	0.98	0.94
SECV	3.16	1.74	3.27
bias	0.08	0.04	0.03
slope	0.97	0.98	0.96
LV	6	2	3
MIR			
R^2^_CV_	0.94	0.91	0.92
SECV	6.68	8.04	4.23
bias	−0.08	0.03	−0.08
slope	0.90	0.92	0.92
LV	5	3	1

ALL: all samples; CPF: chickpea flour; FxMF: flaxseed meal; MIR: mid-infrared; NIR: near-infrared; SECV: standard error in cross-validation; LV: latent variables; R^2^_CV_: coefficient of determination of cross-validation.

## Data Availability

Data are contained within the article.
